# Beef Cattle on Pasture Have Better Performance When Supplied With Water Trough Than Pond

**DOI:** 10.3389/fvets.2021.616904

**Published:** 2021-04-29

**Authors:** Gabriela Schenato Bica, Luiz Carlos Pinheiro Machado Filho, Dayane Lemos Teixeira

**Affiliations:** Laboratório de Etologia Aplicada, Departamento de Zootecnia e Desenvolvimento Rural, Universidade Federal de Santa Catarina, Florianópolis, Brazil

**Keywords:** water intake, average daily gain, beef cattle welfare, extensive pasture, behavior

## Abstract

The behavior and performance of steers on pasture regarding water availability in troughs or in ponds were compared. Eight paddocks were randomly allocated to one treatment: POND (~30 m of diameter) or TROUGH (water trough, 120 cm diameter and 60 cm high and 500 L capacity). Eight groups of six beef steers were randomly assigned to one of the paddocks. The first 10 days were considered for animal habituation. Animals were individually weighed (days 0, 30, 60, and 90). Beginning in the day after each weighing on days 30 (Month 1), 60 (Month 2), and 90 (Month 3), behavior and animal distribution in the paddock were recorded by direct visual observation in three periods of 4 consecutive days. Water temperature and fecal and herbage DM were also recorded in these periods. Water intake was measured during 16 random days in the troughs. Data were analyzed using Generalized Linear Mixed Models, with treatment and period as fixed effects. TROUGH steers gained more weight (0.44 vs. 0.34 kg/day/animal; *P* ≤ 0.007) during the experiment and were heavier than the others at the end of the study (*P* ≤ 0.05). POND steers spent more time drinking water, but TROUGH steers increased the number of drinking events throughout the study (*P* ≤ 0.05), suggesting an adaptation for the new type of water source. Both treatments increased grazing time throughout the study, but not ruminating time (*P* ≤ 0.05). Walking time differed between treatments in all periods of behavior observation (*P* ≤ 0.05). Events of animal licking and ingesting salt of POND steers reduced throughout the study (*P* ≤ 0.05). The number of drinking events of TROUGH steers increased throughout the study, and drinking events were longer for POND steers than TROUGH steers (*P* ≤ 0.05). TROUGH steers spent more time on pasture on Month 2 (*P* ≤ 0.05). Period collection did not affect the water intake of TROUGH treatment (*P* > 0.05). This study demonstrates that water available in troughs rather than ponds for steers on pasture has positive effects on their weight gain and affects cattle behavior.

## Introduction

Water supply and presentation for bovines have been subjects of increasing interest as they affect cattle drinking behavior and preference (1–4). Water quality also affects grazing behavior and cattle performance (5). The lack of appropriate water supply may lead to water restriction, with detrimental effects to water consumption and animal welfare (6–8) as well as animal performance (5, 9, 10).

Dry matter ingestion and cattle performance are closely related to water consumption. Restricted water intake reduces feed intake (11, 12) and may result in lower weight gain. The need of water for appropriate growth, reproduction, digestion, excretion, and all body processes and metabolism is well-known (13). Water required by cattle is achieved from the sum of that intake from plants and other feeds, plus that consumed as free water (13), with the later being usually the major part of the water consumed. Water is perhaps the cheapest nutrient to offer in most production systems, and certainly the most important, affecting directly all body functions. Nevertheless, allocation of water to cattle is mostly underestimated, especially in situations where beef cattle are raised on pasture.

To assure adequate water ingestion for beef cattle in pasture-based systems, a maximum of 250-m walking distance from the water source is recommended (14). However, this recommendation depends on the paddocks size and their arrangements as water source location and water availability that can influence the distance that animals travel in search for water and the number of visits to the water trough. If water is outside of small paddocks, e.g., in the corridor, even a 150-m distance will affect the number of visits, water consumption, and access of subordinate animals (7). A water source located inside the paddock has positive effects on animal performance (15).

The use of water troughs decreases the energetic demand of animals to find a water source (16) and is a useful management strategy to improve the distribution of animals in pastures in order to preserve natural water sources (17), as off-stream watering generally shifts cattle drinking from river to water (18). However, careful placement is required to improve the likelihood that cattle will find and use these water sources and thus decrease their dependence and use of permanent streams and associated riparian areas (19).

Furthermore, it provides availability of water in adequate quantity and quality to the animals. Any low manure contamination in the water from the pond can affect water intake. Dairy cattle can detect low levels (as 0.005% in the water) of manure contamination in their drinking water, avoiding drinking it whenever is possible (20). Clean water available in a trough instead of pond water pumped to a trough or direct access into the pond resulted in an increase of 23% in yearling heifer performance (9). Among different water troughs, cattle may have preferences. They prefer and drink more water from a round plastic than from a squared concrete trough (4). Likewise, they prefer and drink more water from larger than from smaller troughs (2) and deeper and wider to shallow water troughs (3).

There is a growing concern regarding the environmental impact of cattle accessing natural water sources (21, 22). In fact, when having the choice beef cattle would prefer to drink in a water trough than in a natural stream (1). In that study, however, cattle performance was not evaluated. Perhaps the scarcity of information on water source and beef performance is one of the reasons why the vast majority of cattle on pasture drink water from streams, rivers, lakes, or ponds. Farmers usually consider enough having any natural source of water for beef cattle and are not aware of any effect on cattle performance, drinking behavior, or welfare, regarding the source of water. This is largely the reality of cattle on pasture in all countries. With the aim of bringing information on that issue, this experiment was designed to compare the behavior and the performance of beef steers reared in a continuous grazing system, regarding water availability in troughs or in ponds.

## Materials and Methods

The experiment was carried out in a private farm (Cacupé Farm) in the municipality of São Gabriel, the South of Brazil, at the geographic location of 30°20′S and 54°19′W, with an average altitude of 124 m. It was carried out from January to April of 2005, when air temperature ranged from 17.1 to 31.1°C, and total rainfall was 165 mm along the 4 months. Before the beginning of the experimental period, all animals were kept under a continuous grazing system (200 ha) with *ad libitum* mineral salt and water from natural ponds. The pasture was mostly composed by native species, as Uruguayan rice grass (*Piptochaetium montevidensis*), Spanish clover (*Desmodium incanum*), strongback (*Desmodium adscendens*), rescue grass (*Bromus catharticus*), cane grass (*Eragrostis plana*), Australian jointvetch (*Aeschynomene falcata*), and beard grass (*Andropogon bicornis*).

### Study Description

The area of the experiment (32 ha) was equally divided into eight paddocks, with similar pasture botanical composition (as described), natural shade (trees), and mineral salt mix offered *ad libitum*. The eight paddocks were randomly allocated to one of the treatments: POND treatment, four paddocks had water available in a pond of ~30 m of diameter. TROUGH treatment, four paddocks had water available in a round water trough made of polythene (120 cm diameter and 60 cm high and 500 L capacity; Tigre®, Joinville, SC, Brazil). The water from one of the ponds was pumped to a 2,000-L reservoir and then distributed by gravity to the water troughs. A floating ball controlled the water level of the troughs.

A total of 48 beef steers, crossbred of Nelore and Hereford, with average age of 15 months and weighting 189.1 ± 35.35 kg were used. Animals were blocked by body weight and randomly allocated to one of the eight groups of six. Then, the groups were randomly assigned to one of the eight paddocks. All animals were identified by ear tags and coat color and were individually marked with numbers on their sides with black livestock markers (Raidex®, Dettingen; Erms, Germany). Animals from both treatments had no experience with the water trough before the study.

### Measurements

#### Weight Gain and Dry Matter Intake

Animals were individually weighed (individual scale CAUDURO 40100−1,500 kg, Cachoeira do Sul; Brazil) located next to the paddocks at the beginning of the study (Day 0) and on days 30, 60, and 90, always at 9:00 h, after 3 h of fasting. The average daily gain (ADG) was determined by the difference between weights on Day 30, Day 60, and Day 90 divided by the number of days between each measurement (i.e., 30).

#### Behavior and Distribution

Animal behavior was directly recorded in three periods of 4 consecutive days (named Month 1, Month 2, and Month 3), starting in the day after each weighing on days 30, 60, and 90. In the first 2 days of each period, two groups of each treatment were observed simultaneously from 6:00 to 12:30 h on day 1 and from 12:30 to 19:00 h on day 2. On days 3 and 4, the same procedure was made with the remaining two groups of each treatment. That is, each group was observed for 13 h per period. Four observers watched different groups simultaneously. The observers were trained before the study to ensure interobserver reliability (23), and they were balanced across groups and treatments, in order that every observer recorded equally both treatments. The four observers were the same throughout the entire study.

Behaviors were recorded every 10 min using the instantaneous scan sampling technique (23, 24). The behaviors observed were grazing (animal with the mouth below or at the level of the forage or grabbing forage, may be stationary or moving forward), ruminating (animal chewing with lateral jaw movements with the head at the same level or above its body, lying or standing), walking (animal moving, with the head above the superior level of the forage), and other (any other behavior not described above, such as mineral salt and water ingestion and interacting with other animals), according to the definitions adopted by the Laboratory of Applied Ethology and Animal Welfare (LETA) of the Federal University of Santa Catarina (7).

All events of animal licking and ingesting salt were recorded by continuous observation. The number and duration of drinking bouts were also recorded. All events of drinking (i.e., drinking bout) were defined as the beginning to the end of submerging lips in water with perceivable swallowing movements at the throat.

Location of the animals in the paddock (shade, pasture, or water source) was recorded every 20 min. The animal was considered in the shade when the head and most of its body was covered by the shade; at the water source, when standing or lying at <5 m from the water source.

#### Water Intake, Water Temperature, and Climatic Parameters

Daily water intake was measured during 16 random days of the experiment (on days 32, 39, 40, 45, 51, 52, 60, 61, 65, 66, 67, 68, 79, 85, 86, and 87). Only water intake from TROUGH treatment was recorded. During these days, the volume of water required to fill the trough in 24 h was measured using a flow meter (Tecnobrás®, Brazil; precision of 0.01 L) attached to the water inlet.

During the behavior observation days, the temperature of water from ponds and troughs was measured every 2 h from 7:00 to 19:00 h, using floating thermometers (Dolphin®, Guangdong, China) submerged at ~4 cm under water surface. If an animal was drinking water at the same moment of water temperature measurement, the observer waited until the animal gets a distance from the water source. Daily climatic parameters were obtained from the Meteorological Station of *Fundação Estadual de Pesquisa Agropecuária* (FEPAGRO) in the city of São Gabriel.

#### Dry Matter of Fecal and Herbage Sampling

The collection of fecal samples was carried out between 7 am and 9 am of the following day after the behavior observations, with the animals in their respective paddocks. The sample was collected close to the soil, immediately after defecation, disregarding the bottom and top parts. Fecal samples were placed in identified sterile plastic bags and were stored at −18°C. To obtain the DM content, the samples were placed in an aluminum tray, weighed and oven dried at 100°C for 48 h, and then weighed again.

Right after the collection of fecal sampling, pasture sampling was carried out. Five herbage samples of 0.25 m^2^ were randomly collected from each paddock (25). The samples were cut close to the ground and immediately placed in identified sterile plastic bags and stored at −18°C. To obtain DM contents of herbage, samples were placed in identified paper bags and dried in forced circulation oven at 60°C for 48 h (26).

### Statistical Analysis

Descriptive statistics were calculated using Microsoft® Excel® for Windows. Data from water intake of TROUGH treatment measured during 16 random days were grouped into two periods, where Months 1–2 covered data collected from days 32 to 59 and Months 2–3 covered data collected from days 60 to 87. The total amount of water drank during 24 h in each paddock was divided by 6 to achieve an average of water consumption/animal/day. ADG, final body weight, water temperature, water intake, and DM of fecal and herbage data were analyzed using Generalized Linear Mixed Models (Proc Glimmix) of Statistical Analysis Software (SAS) 9.3. Models included treatment and period as fixed effect. The interaction between treatment and period was removed from the models as it was not significant (*P* > 0.10). In the models for ADG, final body weight, and DM of fecal, animal was used as the experimental unit. In models for water temperature, water intake, and DM of herbage, paddock was used as the experimental unit. For water intake and DM of herbage, gamma was included as the type of distribution.

The frequency of grazing, ruminating, walking, and other behavior; the frequency of position of the animal in the paddock (shade, pasture, or water source); the frequency of events drinking water and licking and ingesting mineral salt; and the duration of drinking bouts were also analyzed using Generalized Linear Mixed Models (Proc Glimmix) of SAS. Models included treatment and period as fixed effect. With exception of ruminating behavior and the duration of drinking bouts, interactions between treatment and period were included in the models as they were significant (*P* ≤ 0.05). Animal within paddock was used as the experimental unit, and gamma was included as the type of distribution. Results are reported as least square means (LSM) with the associated standard error of means (SEM). Statistical differences are reported when *P* ≤ 0.05, and tendencies were reported when 0.05 < *P* ≤ 0.10.

## Results

### Weight Gain and Dry Matter Intake

TROUGH steers had higher (29%) ADG than POND steers (*P* = 0.007; Table 1). ADG was lower on Month 3 than Month 1 and Month 2 (*P* ≤ 0.0001; Table 1) in both treatments. Despite that the initial body weights were similar in all groups (189.1 ± 35.35 Kg; *P* > 0.05), TROUGH steers (228.2 ± 1.50 kg) were heavier than POND steers (219.4 ± 1.50; *P* ≤ 0.0001) at the end of the study.

**Table 1:** Effect of treatment and period on ADG, water temperature, and DM of fecal and herbage sampling (LSM ± SEM).

	**Treatment**			**Period**				**Statistics**		
	**POND**	**TROUGH**	**s.e.m**.	**Month 1**	**Month 2**	**Month 3**	**s.e.m**.	**Treatment**	**Period**	**Treatment × period**
ADG* (kg/day)	0.34*y*	0.44*x*	0.037	0.47*a*	0.50*a*	0.21*b*	0.036	≤ 0.007	≤ 0.001	NS
Mean water temperature (°C)	28.7	29.3	0.46	29.7	28.1	29.2	0.57	NS	NS	NS
DM of fecal (%)	17.6*x*	16.1*y*	0.26	16.4	17.0	17.1	0.30	≤ 0.0001	NS	NS
DM of herbage (%)	45.5	47.7	1.40	42.6*b*	46.3*b*	51.3*a*	1.72	NS	≤ 0.01	NS

*NS, non-significant; ADG, average daily gain; DM, dry matter; s.e.m, standard error of means. Different letters “a, b” represent significant differences (P ≤ 0.05) between periods of observation. “x, y” represent significant differences (P ≤ 0.05) between treatments*.

### Behavior and Distribution

**Figure 1:**
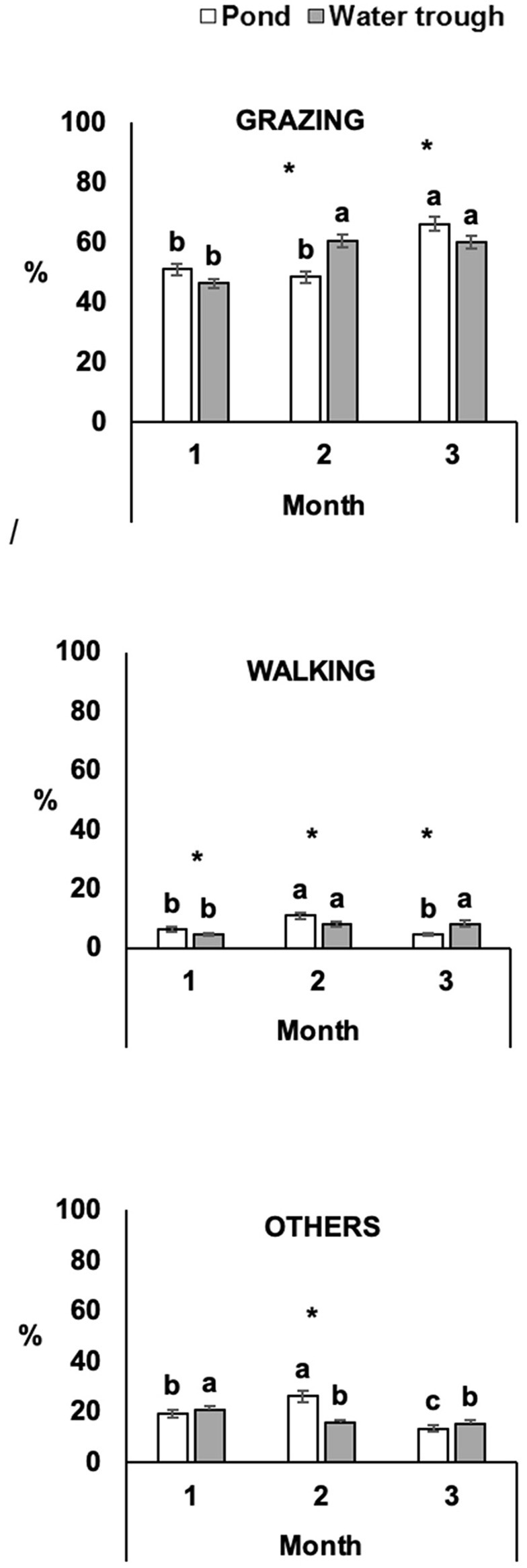
Effect of treatment and period on steer behavior (least square means ± s.e.m.). Grazing, walking, and other behaviors are expressed in least square means ± s.e.m. of the percentage of animals performing each behavior on the three periods of observation. Different letters “a, b, c” represent significant differences (*P* ≤ 0.05) between periods of observation within treatment. “*” represents significant differences (*P* ≤ 0.05) between treatments within the period of observation.

Treatment did not affect the ruminating time (*P* > 0.05), but it was lower on Months 2 (18.4 ± 0.87) and 3 (20.0 ± 0.94) than on Month 1 (27.2 ± 1.28; *P* ≤ 0.05). For grazing, walking, and other behavior, there were interactions between treatment and period of observation (*P* ≤ 0.05; Figure 1). On Month 2, TROUGH steers spent more time grazing than POND steers (*P* ≤ 0.05); in contrast, POND steers spent more time grazing than TROUGH steers on Month 3 (*P* ≤ 0.05). Both treatments increased grazing time throughout the study (*P* ≤ 0.05). Walking time differed between treatments in all periods of behavior observation (*P* ≤ 0.05). Within treatment, walking time was higher on Months 1 and 2 for POND steers, while it was lower on Month 3 (*P* ≤ 0.05). Other behaviors, which included any other behavior not previously mentioned, such as mineral salt and water ingestion and interaction between animals, differed between treatments in Month 2 (*P* ≤ 0.05). Within treatments, POND steers spent more time performing other behaviors on Month 2, while TROUGH steers spent more time in other behaviors on Month 1 compared to the other periods of behavior observation (*P* ≤ 0.05).

**Figure 2:**
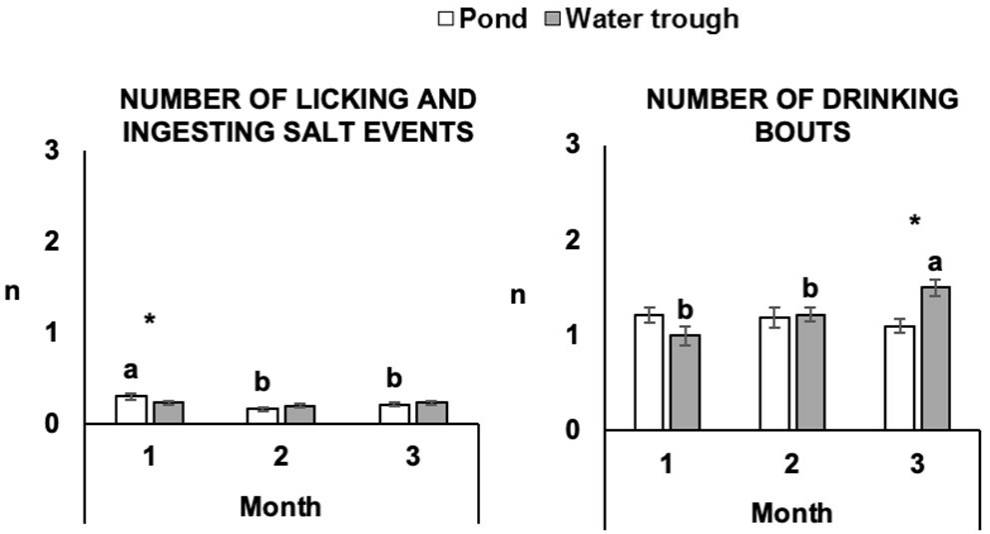
Effect of treatment and period on the number of events of animal licking and ingesting salt and number and duration of drinking bouts. The number of events of animal licking and ingesting salt and drinking bouts is expressed in least square means of the number of events per animal per period of observation. The duration of drinking bouts is expressed in least square means of the duration (s) of events per animal per treatment or period of observation. Different letters “a, b” represent significant differences (*P* ≤ 0.05) between periods of observation within treatment. “*” represents significant differences (*P* ≤ 0.05) between treatments within the period of observation.

There were interactions between treatment and period of behavior observation on the number of events of animal licking and ingesting salt and number of drinking bouts (Figure 2). The number of events of animal licking and ingesting salt of POND steers was higher on Month 1 than Months 2 and 3 (*P* ≤ 0.05), while it remained constant for TROUGH steers (*P* > 0.05). In Month 3, animals in the TROUGH treatment had a higher number of drinking bouts than in the POND treatment (*P* ≤ 0.05). The number of drinking bouts of TROUGH steers increased throughout the study (*P* ≤ 0.05), while it remained constant for POND steers (*P* > 0.05). There was no interaction between treatment and period of behavior observation on the duration of drinking bouts (*P* > 0.05). However, drinking bouts were longer in POND steers (59.2 ± 3.32) than TROUGH steers (43.5 ± 3.19; *P* ≤ 0.001) and both treatments had longer drinking events in Month 1 (65.9 ± 4.49) than Month 2 (47.6 ± 3.73) and Month 3 (40.5 ± 3.45; *P* ≤ 0.001).

**Figure 3:**
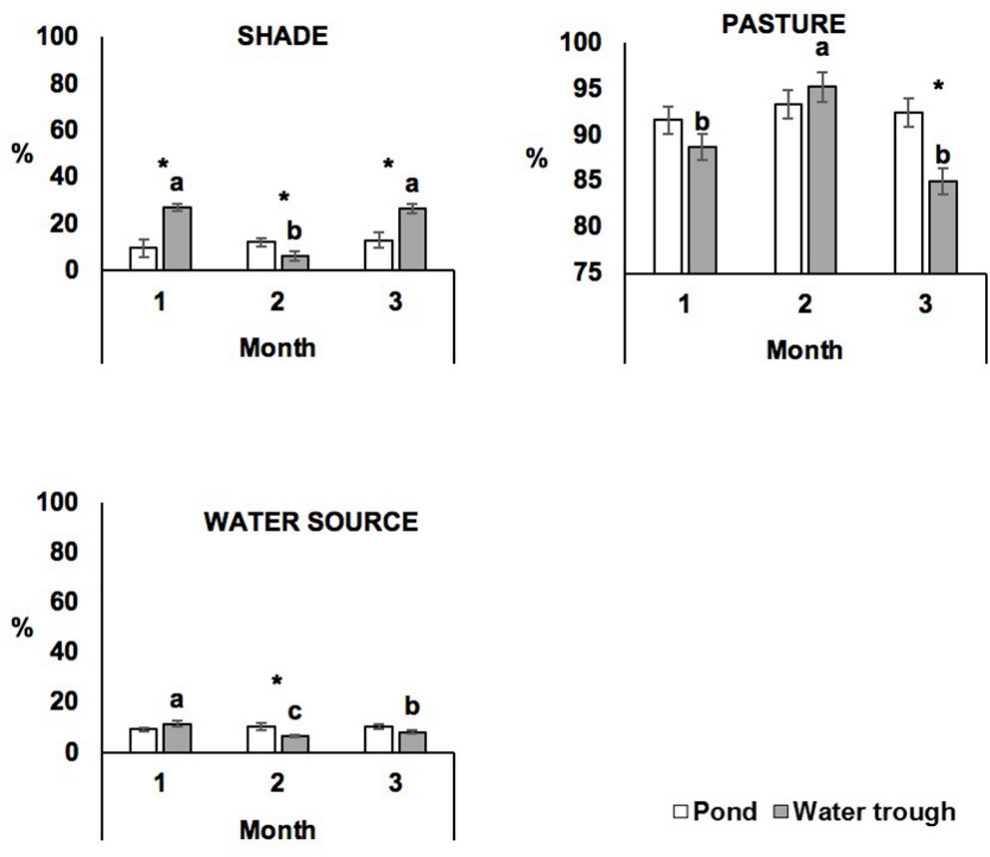
Effect of treatment and period on steer distribution (least square means ± s.e.m.) on the paddock. Shade, grassland, and water source are expressed in least square means ± s.e.m. of the percentage of animals positioned on the three periods of observation. Different letters “a, b, c” represent significant differences (*P* ≤ 0.05) between periods of observation within treatment. “*” represents significant differences (*P* ≤ 0.05) between treatments within the period of observation.

The effect of treatment and period on animal distribution on paddock is presented in Figure 3. POND steers spent a similar time on shade during the three periods of behavior observation, which was similar for time spent on pasture and near to water source (*P* > 0.05). In contrast, TROUGH steers spent more time on pasture on Month 2, which was balanced with less time on shade and water source in this respective month (*P* ≤ 0.05).

### Water Intake, Water Temperature, and Climatic Parameters

Period of data collection did not affect the water intake of TROUGH treatment (*P* > 0.05). During the recording days, TROUGH steers drank 12.5 ± 0.87 L/animal/day (*P* > 0.05).

Averages of minimum and maximum air temperature were calculated for the months of January (min 19.6 ± 2.5°C; max 31.1 ± 2.6°C), February (17.5 ± 1.6°C; max 29.8 ± 2.1°C), March (17.1 ± 3.5°C; max 28.3 ± 4.3°C), and April (17.7 ± 2.4°C; max 29.3 ± 2.1°C). Total rainfall during the 4 months was 165 mm, distributed in 21 days (January: 22.4 mm/5 rain days; February: 33.5 mm/4 rain days; March: 29.5 mm/4 rain days; April: 80.2 mm/8 rain days).

Minimum water temperature was not affected by treatment; however, it was lower on Month 2 than on Month 1 and Month 3 (*P* ≤ 0.001, Table 1). In contrast, period did not affect the maximum water temperature, although it was higher on the TROUGH than on the POND treatment (*P* ≤ 0.05; Table 1). Average water temperature was not affected by treatment or by period (29.0 ± 0.49°C; *P* > 0.05; Table 1).

### Dry Matter of Fecal and Herbage Sampling

TROUGH steers presented lower fecal DM than POND steers (*P* ≤ 0.0001; Table 1), without effect of period or interaction on fecal DM (*P* > 0.05). In contrast, herbage DM was not affected by treatment (*P* > 0.05) but by period, being higher on Month 3 than on Months 1 and 2 in both treatments (*P* ≤ 0.01; Table 1).

## Discussion

The findings of this study showed that water supplied in a trough rather than in a pond has positive effects on animal performance and behavior. Although having the same body weight at the beginning of the study, TROUGH steers had an ADG 29% higher than POND during the experiment and were 4% heavier at the end of the study. Our findings confirm the previous study showing that cattle drinking clean water delivered to a trough gained 23% more weight than cattle drinking from a dugout (9). Similarly, yearling heifers gained 23% more weight when drinking clean water delivered to a trough than those drinking directly from a pond and 20% more than those drinking pond water pumped to a trough (5). Cows and calves with off-stream water also gained more weight than no-off stream animals (27). Brew et al. (28) reported that water intake of beef cattle is positively correlated with feed intake and ADG, which are in line with our findings as steers from both treatment reduced ADG and drinking time throughout the study.

Despite that grazing time was longer on POND treatment on Month 3, the difference of final body weight between treatments may be due to the fact that TROUGH steers increased their grazing time from Month 2, while POND steers only showed an increase on Month 3 compared to the beginning of the study. A previous study also reported an effect of water source on grazing time: cattle with access to clean water also spent more time grazing and less time resting than animals receiving pond water pumped to a trough and those with direct access into the pond (5). In our study, a systematic approach to ensuring similar pasture availability and consumption was not carried out, so it was not possible to affirm that nutrition did not influence weight gain.

DM intake is the most important factor for water intake in bovines (29), followed by milk production, sodium intake, and high temperatures (6). Earlier studies reported a positive correlation between water intake and DM intake (6, 29–31) and salt ingestion (6, 32). Animals under water deprivation have a reduction in food consumption and an increase in urine concentration (33). In contrast, cows accelerate drinking water intake to excrete a large amount of potassium and nitrogen into urine in excess of their needs (34). Despite that DM of herbage mass increased throughout the study in both treatments, the water intake from the TROUGH steers was not affected by the period of the study. Paddocks were set up on the same pasture, and the herbage content of both treatments was equal, as shown in Table 1; therefore, the higher fecal DM of POND steers could indicate that this group had a lower water intake than TROUGH steers. Moreover, fecal DM did not follow the increase in DM of herbage between periods, suggesting an effect of treatments on the moisture content of the feces.

The duration of drinking bouts was longer in steers with access to POND than in TROUGH. Besides that, the number of drinking bouts increased on steers with access to TROUGH in the following periods, being higher than POND in the last period of behavior observation. It has been shown, in a number of experiments reported in a systematic review, that an increased frequency of drinking water resulted in increased weight gain in beef cattle and milk production in dairy cows (35), as was found in this experiment. The increase in the number of drinking bouts of steers with access to TROUGH in this study could be due to animal adaptation to the new type of water source. In the Bagshaw et al. (36) study, beef cattle in the grazing system also increased the use of trough over time.

Water intake by cattle can be affected by other factors including weather conditions, water quality, and height of the water trough. Increased THI (temperature–humidity index) resulted in cows drinking more water, spending more time at the drinker, making more visits to the drinker, and competing more at the drinker (37). Among other characteristics, cattle can discriminate for and select water based on organic solid contents (38) and they reduce water intake due to suspended particulate matter that can influence its appearance, odor, taste, and physical and chemical properties (39). Also, they have an aversion to drink water containing feces (5). In the case of the present study, animals could enter in POND water. Therefore, it was prone to manure contamination from both erosion of the soil adjacent and direct defecation and urination into the water by drinking animals (40). However, despite that suspended particulate matter could be higher on POND treatment, the chemical composition and microbiological quality of water from both treatments did not differ, as water of the troughs was pumped from the pond.

While in POND treatment water was offered at the ground level, in TROUGH it was 60 cm high. In the study of Machado Filho et al. (2), the cows drank more water from a larger and higher trough, also 60 cm high, when they had access for 24 h than on the smaller version (30 cm height). Likewise, beef heifers preferred and drank more from a 60-cm-height round plastic trough than from a 50-cm-height squared concrete trough (4), and dairy cows took more sips, spent more time drinking, and drank more water from higher (60-cm) and larger troughs than small ones (30-cm-height) (3, 41).

The current study was conducted during the hottest months of the summer, under high temperatures, and the average of maximum air temperatures was above 28°C. Temperature and humidity have a direct relationship with cattle water consumption (32, 37, 42). Month 2 of behavior observation was the month with the lowest minimum and maximum air temperatures compared to the other 2 months. Apparently, differences in weather conditions among months did not change POND steers' spatial distribution. However, in Month 2 TROUGH steers spent less time near the shade or water source, therefore spending more time on pasture. In fact, TROUGH steers grazed longer in Month 2, therefore spending more time on pasture. Conversely, in Month 3 POND steers spent more time on pasture and less time in shade than TROUGH steers, and they also grazed longer in this period. These results are unlikely to be explained by the weather conditions but might be related to water intake, once dry matter intake is closely related to water (13).

The availability of shade is essential for grazing animals, and their absence can reduce their well-being (43) and modify their behavior; that is, cows can spend more time around the water source when the shade is unavailable or insufficient (44). In addition, the quantity of forage in the field is likely to alter cattle behavior around water sources (36). In the present study, it was not possible to control the distance between the shade and the water sources (pond or trough), but this could explain the difference between treatments on their spatial distribution throughout this study. The location of the trough seems to be one of the critical factors affecting drinking behavior by cattle (7, 36).

Mean water temperature did not differ between TROUGH and POND treatments. Therefore, the differences found in this study are unlikely to be due to water temperature. Previous studies have reported that beef cattle drink more warm than cold water (45). This finding was later confirmed on dairy calves (46), lactating dairy cows (47), sheep (48), and goats (49).

## Conclusion

This study demonstrates that water available in trough rather than ponds has positive effects on steer performance and affects beef cattle behavior. Steers supplied with water on trough gained more weight and were heavier than the other group of animals at the end of the study. In general, POND steers spent more time drinking water but TROUGH steers increased the number of drinking events throughout the study, suggesting an adaptation for the new type of water source.

## Data Availability Statement

The raw data supporting the conclusions of this article will be made available by the authors, without undue reservation.

## Ethics Statement

Ethical review and approval was not required for the animal study because at the time of fieldwork, Brazil didn't have any regulation requiring previous evaluation from Ethics Committee for non-invasive studies. Even though the activities of the experiment followed conduct respectful to the welfare of the animals involved according to Brazilian Federal Law No. 11.794 of October 8, 2008, later approved, which regulates use of animals in research. Animals were individually weighed following the routine of the farm, and the only direct intervention was marking animals using nontoxic markers for identification in the field. The other activities during the experiment were restricted to visual observation. Written informed consent was obtained from the owners for the participation of their animals in this study.

## Author Contributions

LP and GB conceived, planned, designed the experiment, and wrote the manuscript. GB and DT made the data collection and fieldwork. DT performed statistical analysis and contributed to the manuscript. LP, GB, and DT interpreted data. All the authors approved the final version of the manuscript.

## Conflict of Interest

The authors declare that the research was conducted in the absence of any commercial or financial relationships that could be construed as a potential conflict of interest.
